# An Overview of the Cellular Mechanisms of Flavonoids Radioprotective Effects

**DOI:** 10.15171/apb.2020.002

**Published:** 2019-12-11

**Authors:** Mahdi Mashhadi Akbar Boojar

**Affiliations:** ^1^Student Research Committee, Baqiyatallah University of Medical Sciences, Tehran, Iran.; ^2^Department of Pharmacology and Toxicology, Faculty of Pharmacy, Baqiyatallah University of Medical Sciences, Tehran, Iran.

**Keywords:** Ionizing radiation, Radio-protector, Flavonoids

## Abstract

Considering the remarkable application of radiotherapy in the treatment and diagnosis of various diseases and even nuclear war, it is important to protect healthy tissues and people at risk from the radiation. Currently, there is no ideal and safe radioprotective agent available and we are seeing a great effort to find these agents from natural sources. Phenolic compounds, as well as flavonoid, are presented widely as the second metabolite in plants and they have been considered for investigation according to their benefits for human health, healing and preventing many disorders. The major bioactive benefits of flavonoids include antioxidant, anti-inflammatory, anti-tumor, anti-aging, anti-bacterial and viral, neuroprotection and radioprotective effects. Their lower toxicity and oral administration have made it suitable for radiotherapy patient, radiation, military forces, and even the general public. This review attempts to provide a summary of the main molecular mechanisms involved in flavonoid radio-protective effects. Data of these studies will provide a comprehensive perspective to flavonoids and can help to optimize their effects in radioprotection procedures.

## Introduction

During recent years, along with the development of science and technology, the ionizing radiations are widely used in various fields, including medical diagnosis, therapeutic interventions, and agricultural and industrial sciences.^1^ Hence, humans have been exposed to high levels of radiations in comparison to the past.^2^ In addition, increasing the likelihood of terrorist attacks on power plants and reactors and even unintentional incidents and accidental leakage of radioactive substances have always been a real concern.^3^

Radiation therapy is one of the most common and alternative surgical methods used to treatment of cancer alone or in combination with chemotherapy. Healthy tissue damage and the risk of developing new malignancies are one of the major challenges of radiotherapy that can be prevented by means of radiation protection (or radiological protection) and increased resistance of natural cells to the event.^4,5^

Ionizing radiation beams are responsible for the excessive generation of free radicals in the human body and these radicals play a serious role in the pathogenesis of various diseases, as they are capable of inducing damage to cellular structures and DNA, oxidizing proteins, or inducing peroxidation of lipids.^6^ These destructive effects can lead to protein dysfunction in the hematopoietic system and immune cells, accelerated aging and expanded cellular degeneration.^7^ Therefore, the importance of protecting human beings from radiation in radiotherapy and individuals at risk, especially the military, has increased.^8^

Flavonoids are a group of polyphenolic compounds that so far have been identified more than 9000 of them in different species in many plants.^9^ Since the early 1920s, the bioavailability and other biological effects of flavonoid derivatives have been noted, including the elimination of free radicals, increased resistance to beams, reduced inflammation, anti-tumor effects, and decreased senescence process of cells.^10-12^ Currently, many studies focus on the effects of radioprotective substances. Therefore, it is necessary to examine the radiation protection mechanisms of flavonoids and their proper use in the prevention of possible damages before and after exposure to radiation hazards.

## Materials and Methods

In this review study, related and considerable research papers and review articles of online databases include PubMed, Scopus, Google Scholar and Web of Science which recently published by reliable publishers such as Elsevier, Springer and PLoS One, up to April 2019 were collected and discussed. The literature was searched using the following keywords: Ionizing radiation, Radio-protector, Flavonoids.

### The biological damages induced by ionizing radiation

Naturally, ionizing radiation appears in various forms, such as X-ray, alpha, beta, gamma, and neutron.^13^ Human exposure to these beams can lead to a series of biochemical and pathological changes and ultimately cause injury or necrosis of the target organ.^14^

Current studies show that the damage caused by these beams directly or indirectly affects the human body. Generally, direct damage occurs when large biological molecules in the living organism interact with the radiation physically and acute biological destruction happens.^15^ Indirect damage is mainly due to the production of free radicals that are generated from water molecules in the body.^16^ The ionizing radiation leads to the production of free radicals such as hydroxyl and superoxide radicals that can interfere with the body’s biological macro-molecules and cause significant changes in their structure and function, which, in turn, causes serious injuries in response to the beams.^17^

Damage to the DNA and the immune and hematopoietic systems are the major injuries that the ionizing radiation brings to the living organism. Damage to cellular DNA is involved in the loss of biological information and degradation of normal cellular function, which can consequently lead to the death of cells or escape from the regulating mechanisms of the cell cycle and finally development of cancer.^18^ Immune system and hematopoietic cells are highly susceptible to ionizing radiation. Ionizing radiation can reduce the number of immune cells and weaken their specific and non-specific functions.^19^ In the hematopoietic system, these beams can also reduce red blood cells during the development of bone marrow suppression.^18,20^

### Classification of flavonoids

**Figure 1:**
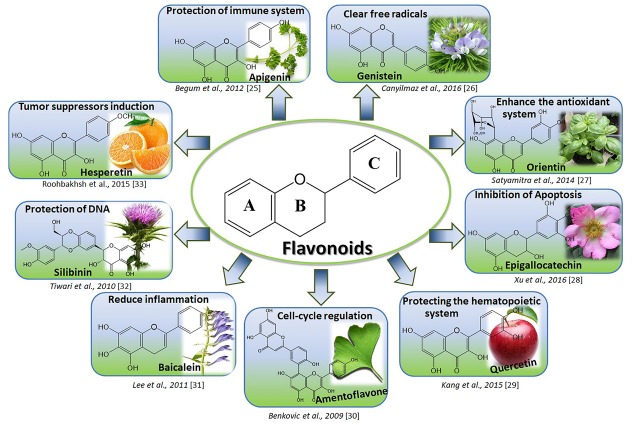
The backbone of the flavonoids chemical structure and the main molecular mechanisms in the radiation protection effects of nine well-known flavonoids.^25-33^

Flavonoids are the major group of polyphenolic compounds with phenyl chromium backbone. These compounds generally have two aromatic rings having a phenolic hydroxyl substituent (ring A and B) attached to the carbon moiety.^21^ They are containing 15 carbon atoms forming a C6C3C6 structure shown in Figure 1.^22^ These compounds are found extensively in the stem or trunk and also in fruits and generally in the form of secondary metabolites in plants.^23^ Flavonoids are classified according to the chemical features of carbon bonds and the number and position of hydroxyl groups in more than 10 different categories, among which flavones, flavonones, flavonols, flavononols, isoflavones, flavanoles (catechins) and anthocyanidins are more important than others, and in continuation will be discussed.^24^

### Cellular and molecular mechanisms of flavonoids in radio-protection

Natural radio-protectives are non-toxic or low toxic products obtained from natural compounds that can be used before or after exposure to ionizing radiation to reduce radiation damage.^34^ The usefulness of flavonoids in various cases, such as cytoprotective properties, antioxidant, and free radical scavenging effects, antiviral and antibacterial effects, anti-tumor properties in the prevention and treatment of cancer, and their anti-inflammatory effects have been proven in various cellular and animal studies.^35-37^ For example, administration of different flavonoids in mice has significantly increased the survival of 30 days and decreased apoptosis-necrosis induced by ionizing radiation.^38,39^

In general, the main mechanisms of radiation protection for flavonoids include DNA protection, antioxidant activity, immune and hematopoietic systems protection, and inflammation reduction, which are discussed below.

#### Prevention of DNA damage and genotoxicity

DNA is a major target of ionizing radiation. Secondary DNA damage due to the exposure to ionizing radiation can cause many harmful effects on human life. Therefore, reducing the destructive effects of ionizing radiation on DNA is an important problem in protecting the body from ionizing radiation.^40^

Alkaline comet assay to detect the effects of Baicalein on the radiation exposure induced DNA damage showed that the administration of this flavonoid would have a significant protective effect before exposure to the radiation.^41^ Micronucleus assay in rat blood cells exposed to radiation also indicated a reduction in damage to the bone marrow. It has been shown that treating mice lymphocytes exposed to 3 Gy of ionizing radiation with Silibinin reduces DNA damage and microcrystalline formation. Oral administration of Silibinin to mice before the exposure of the animal body to radiation (7.5 Gy), significantly attenuated the death caused by these beams and also ameliorated the blood cells injuries.^32^

The information obtained from the measurement of cytokinesis-block micronucleus in assessing of Apigenin radio-protector effects on human lymphocytes suggests a reduction in the injuries to the chromosome from ionizing radiation.^25^ This flavonoid significantly reduces the rate of micronuclei (MN) formation in a dose-dependent manner.

Xu Ping and colleagues assessed the radiation protection effects of the Guipi pill flavonoids in mice and showed that these flavonoids increased significantly the thirty-day survival rate by administration eight days prior to exposure to ionizing radiation of at least 8% in these animals.^42^ It has been shown that these flavonoids increase the number of white blood cells and protect bone marrow DNA content.^43^

In addition, flavonoids from the families Ocimum (orientin), naringin, procyanidin, the isolated flavonoids from propolis, and *Gentianella* flavonoids can also effectively reduce the genetic toxicity of the beams and protect DNA from the radiation damage.^30,44-46^

### Free radical clearing ability and antioxidant effects

Ionizing radiations can produce a large number of free radicals by affecting the aquatic environment of living organisms. These radicals can reduce the activity of superoxide dismutase (SOD), glutathione peroxidase (GSH-Px), and catalase (CAT), causing lipid peroxidation and an increase in malondialdehyde (MDA) index, which have great potential for damaging the cell membrane and DNA content.^6,41^ Flavonoids can effectively clear oxygen-derived free radicals and remove the indirect effects of ionizing radiations on cells in the human body.^47^ In many animal studies, these effects have been able to diminish the deaths from radiation received.^48^

It has been shown that *Humuluslupulus* flavonoids can induce the activity of SOD, GSH-Px, and CAT and reduce the MDA content in mice exposed to radiation. These flavonoids can also increase the number of blood leukocytes and show a protective effect on the immune system of the mice.^49^ A comparative study of the human keratinocyte cell line HaCaT indicated the high ability of quercetin and the next genistein to purify the free radicals of hydroxyl, induced by UVB rays. In addition, the treatment of the cells with the flavonoids mentioned above before contact with the radiation increased the activity of SOD and reduced the levels of tumor necrosis factor (TNFα), reactive oxygen species (ROS) and MDA.^50^

In a similar study, a significant increase in total antioxidant capacity and elevated SOD activity in the PC12 clone, developed from a pheochromocytoma tumor of the rat adrenal medulla treated with quercetin was quite significant compared to control cells.^51^ It has been demonstrated that the breviscapine flavonoids collection (derived from Chinese herb *Erigeron breviscapus*) effectively neutralizes radiated free radicals and subsequently increases the total intracellular antioxidant capacity by attenuation of lipid peroxidation, thereby contributing to the cytoprotective role from ionizing radiation.^52^

Ping et al evaluated the effects of amentoflavone radiation protection by measuring cell viability, apoptosis, and ROS levels after exposure to 8 Gy gamma rays from ^60^Co in guinea pig pulmonary fibroblast cells. This study confirmed that treatment with this flavonoid in the 24-hour period before exposure significantly inhibited apoptosis and reduced levels of ROS.^39^ Also, another similar study showed that administration of 200 mg/kg of genistein at one hour prior to X-ray exposure improved bone marrow function by up to 44% and elevated the 30-day life expectancy index in mice.^53^

## Protective effects on the immune system

The immune system is extremely sensitive to harmful radiation. Exposure to ionizing radiation can lead to functional impairment of the immune system and even death resulting from a decrease in the number of immune cells. Also, ionizing radiation can cause problems in producing antibodies and impair the regulation of cytokine network.^19^

Among bio-flavonoids, isoflavones, which are often found in Fabaceae (i.e., *Leguminosae*, or bean) family, have a greater role in protecting the immune system against ionizing radiation.^43^ It has been shown that treatment of mice with only 4 Gy to ionizing radiation is sufficient to reduce the immune function of the thymus and the spleen. Mice receiving soy isoflavones significantly improved lymphocyte function index, decreased apoptosis, reduced cells in the G_0_ or G_1_ phase and increased the presence of cells in G_2_ and post-irradiation mitotic cells. In addition, a significant increase in the macrophage recognition and phagocytic ability and serum levels of immunoglobulin, IgA, IgG, and IgM were also observed.^54^ The flavonoids from buckwheat (especially Tartary buckwheat) also have the same effects on the protection of immune cells, especially T lymphocytes.^43^

The literature review reveals that quercetin, apigenin, hesperidin, and rutin can significantly enhance lymphocyte proliferation and secretion of key immune system cytokines and remarkably reduce the damage to peripheral blood lymphocytes.^55-58^ It has been demonstrated that administrating resveratrol to workers who work in an environment with a high potential for electromagnetic hazards can help to reconstruct the expression and function of the nuclear factor-kappa B (NF-kappa B), IL-6, and ultimately prevent the deterioration of the immune system.^59^

### Protective effects on the hematopoietic system

The human hematopoietic system cells are highly sensitive to radiation due to the high volume of proliferation and cell division. Ionizing radiations, target all bone marrow stem cells.^60^ Hence, protecting hematopoietic stem cells is an important factor in preventing radiation damages. There have been several new findings demonstrate that flavonoids can protect bloodstream organs from radiation damage and improve their repair to increase body resistance to radiation injuries.^61^

Synthetic derivatives of tetrahydroxyflavone (such as fisetin) have been shown to possess a more potent biological effect than their natural source. It has been proved that the treatment of mice exposed to gamma rays from ^60^Co with these flavonoids, largely contributes to the regeneration and function improvement of hematopoiesis.^62^

Previous studies have shown similar effects of quercetin, genistein, and soluble derivatives in propolis to prevent the reduction of white and red blood cells, platelets and hemoglobin, as well as damage to leukocyte DNA from ionizing radiation.^63-65^ The pivotal part of these effects seems to be due to increased production of granulocyte colony-stimulating factors as a key factor in the recovery of stem cells.^43^ It has been confirmed that administration of genistein nanoparticles suspensions in mice reduces the death of hematopoietic stem cells from 43% to 77% and decreases suppression of pro-inflammatory factors such as IL-6 and COX-2 in mice bone marrow and spleen cells.^43^

## Reducing effects on inflammation

Major radiation exposure can lead to severe inflammatory reactions. Inflammatory cytokines can cause damage or necrosis in any tissues of the body. Such injuries are more common in the lung and kidneys.^66^

Soybean isoflavones have promising protective effects both before and after exposure to radiation in the lung tissue by reducing the infiltration of macrophages and neutrophils in alveolar and bronchial surfaces and ultimately reducing fibrosis.^67^ Oral administration of *Astragalus complanatus* plant flavonoids after exposing mice to 10 Gy resulted in a significant reduction in the serum transforming growth factor β, TNF-α and IL-6 levels, and consequently reduced inflammatory damage in the exposed mice.^68^

Baicalein, as the most active flavonoid in *Paeonia lactiflora* (Chinese peony or common garden peony), can suppress inflammatory responses from radiation by modulating the NF-kB and increasing the activation of FOXO transcription factors, CAT and SOD in mouse kidney. In addition, this compound inhibits the phosphorylation of mitogen-activated protein kinase (MAPK) and Akt induced by the beam, that amplify NF-kB kinases.^31^ It has recently been shown that administration of quercetin and hesperidin in mice can significantly prevent intestinal damage by decreasing TNF-α levels and enhancing IL-10.^69^

## Flavonoids and apoptosis

In the most cellular and molecular studies of cancerous cells, flavonoids have been characterized as an apoptosis inducer by various mechanisms.^36^ Some pro-apoptotic pathways such as BAD, BID, Bax, caspases 3, 8, and 9, tumor suppressors including p53, cell-cycle inhibitors such as some cyclin-dependent kinases and ceramide activator cascades and its messengers in tumor cells have been enhanced by flavonoids.^70-72,10^ In contrast to the inhibitors of apoptosis, such as phosphoinositide-3-kinase, along with protein kinase B, MAPK, metalloproteinases 2 and 9, and many growth factors that promote tumor cell proliferation and differentiation, were suppressed by flavonoids.^73-75^

However, the effects of flavonoids in healthy and non-cancerous cells have been largely inconsistent with tumor cells. The protective effects of flavonoids on neural cells, liver, kidney, heart, skin and immune and hematopoietic systems have been discussed in numerous investigations.^76-79^ Flavonoids distinctions between these two groups of cells have created a great promise in applying them for radioprotection and other beneficial effects.

## Conclusion

According to the previous studies in this field, flavonoids as natural radio-protector in radiotherapy applications for the protection of healthy cells, unexpected radiation accidents and also in terrorist incidents can be effective for reducing the harmful effects of radiations. Protecting the DNA, immune and hematopoietic systems, clearing free radicals, strengthening the immune response and antioxidant properties of flavonoids, justify these abilities. Although generally, these compounds in mild doses are often not toxic to healthy cells, they have considerable toxicity on tumor cells by induction of programmed cell death. If future clinical trials confirm the radio-sensitizer effect of these compounds to the malignant tissues as well as the protective properties of radiation on healthy tissues, they can also be used as a radio-protector for safety and efficiency in advanced radiation therapy.

## Ethical Issues

Not applicable.

## Conflict of Interest

None.
